# Presentation and treatment of two cases of malignant struma ovarii

**DOI:** 10.1186/s12905-024-03002-5

**Published:** 2024-03-05

**Authors:** Rawan A. Obeidat, Sakhr Alshwayyat, Tala Abdulsalam Alshwayyat, Anwar Rjoop, Qosay Mahmoud Sharqiah

**Affiliations:** 1 Department of Obstetrics and Gynecology, Faculty of Medicine, Jordan University of Science and Technology, P. O. Box: 3030, Irbid, 22110 Jordan; 2 Faculty of Medicine, Jordan University of Science and Technology, Irbid, Jordan; 3 Department of Pathology, Faculty of Medicine, Jordan University of Science and Technology, Irbid, Jordan

**Keywords:** Malignant struma ovarii, Mature cystic teratomas, Radioactive iodine, Salpingo-oophorectomy, Hysterectomy

## Abstract

**Background:**

Malignant Struma Ovarii (MSO) is a rare type of germ cell tumour which is diagnosed postoperatively on surgical pathology specimens by the presence of differentiated thyroid cancer in mature cystic teratomas in the ovaries. Treatment and follow-up procedures are not clearly established due to the paucity of MSO cases.

**Case 1:**

A 44-year-old multiparous female presented with an irregular period. Ultrasound showed a left ovarian lesion mostly a dermoid cyst, however, CT showed a 3.8 × 2.7 × 4 cm complex cystic lesion with thick septation and enhancing soft tissue component. Laparoscopic left salpingo-oophorectomy was performed and histopathology showed a follicular variant of papillary thyroid carcinoma arising in a mature cystic teratoma. Peritoneal cytology was positive for malignancy. A thyroid function test was normal before surgery. Total thyroidectomy was performed followed by radioactive (RAI) iodine therapy. Later, a total laparoscopic hysterectomy and right salpingo-oophorectomy were performed. There is no evidence of recurrent disease during the 26-months follow-up.

**Case 2:**

A 46-year-old single female presented with left lower abdominal pain that had persisted for 2 months. Imaging revealed an 8 × 9 × 9.5 cm left ovarian mass. Laparoscopic left salpingo-oophorectomy was performed and histopathology showed mature cystic teratoma with small papillary thyroid cancer. CT showed no evidence of metastatic disease. Later, the patient had a total thyroidectomy followed by radioactive (RAI) iodine therapy. She was started on thyroxine and later had total abdominal hysterectomy and right salpingo-oophorectomy.

**Conclusion:**

MSO is a very rare tumour. Preoperative diagnosis is very difficult because of the nonspecific symptoms and the lack of specific features in imaging studies. Also, there is no consensus on the optimal treatment of women with MSO. Our two cases add to the limited number of MSO cases.

**Supplementary Information:**

The online version contains supplementary material available at 10.1186/s12905-024-03002-5.

## Introduction

Struma ovarii (SO) is a specialized or monodermal teratoma predominantly composed of mature thyroid tissue [1]. It is recognized by histological examination when more than 50% of the teratoma is occupied by thyroid tissue [2, 3]. SO accounts for approximately 2–5% of all ovarian teratomas [1, 3]. Less than 5% of SO cases present with malignant transformation [3–7]. 

Malignant Struma Ovarii (MSO) is a rare type of germ cell tumour which is diagnosed postoperatively on surgical pathology specimens by the presence of differentiated thyroid cancer in mature cystic teratomas in the ovaries. MSO is most common between the ages of 30 and 40 years [4]. Savelli et al. reported in their study that 42% of MSO cases were asymptomatic [8]. Women with MSO usually present with abdominal or pelvic pain and/or palpable lower abdominal mass [5, 9–12]. Ascites, abnormal menses and /or elevated CA125 are sometimes present [4, 6, 12]. Clinical and biochemical features of hyperthyroidism are uncommon in women with MSO, occurring in less than 5% of cases [1, 7, 9]. 

Due to MSO rarity, there has been some controversy about the diagnosis and treatment of MSO [6]. MSOs are usually treated with local surgery (unilateral salpingo-oophorectomy (USO), or hysterectomy with bilateral salpingo-oophorectomy (BSO). Total thyroidectomy, radioactive iodine ablation (RAI), and thyroid hormone suppressive therapy could be performed based on the presence of metastatic disease, the risk of recurrence and molecular genetic analysis [10, 11, 13]. Presented are two cases of MSO treated with a combination of surgery and radioactive iodine.

## Clinical cases

### Case 1

**Fig. 1:**
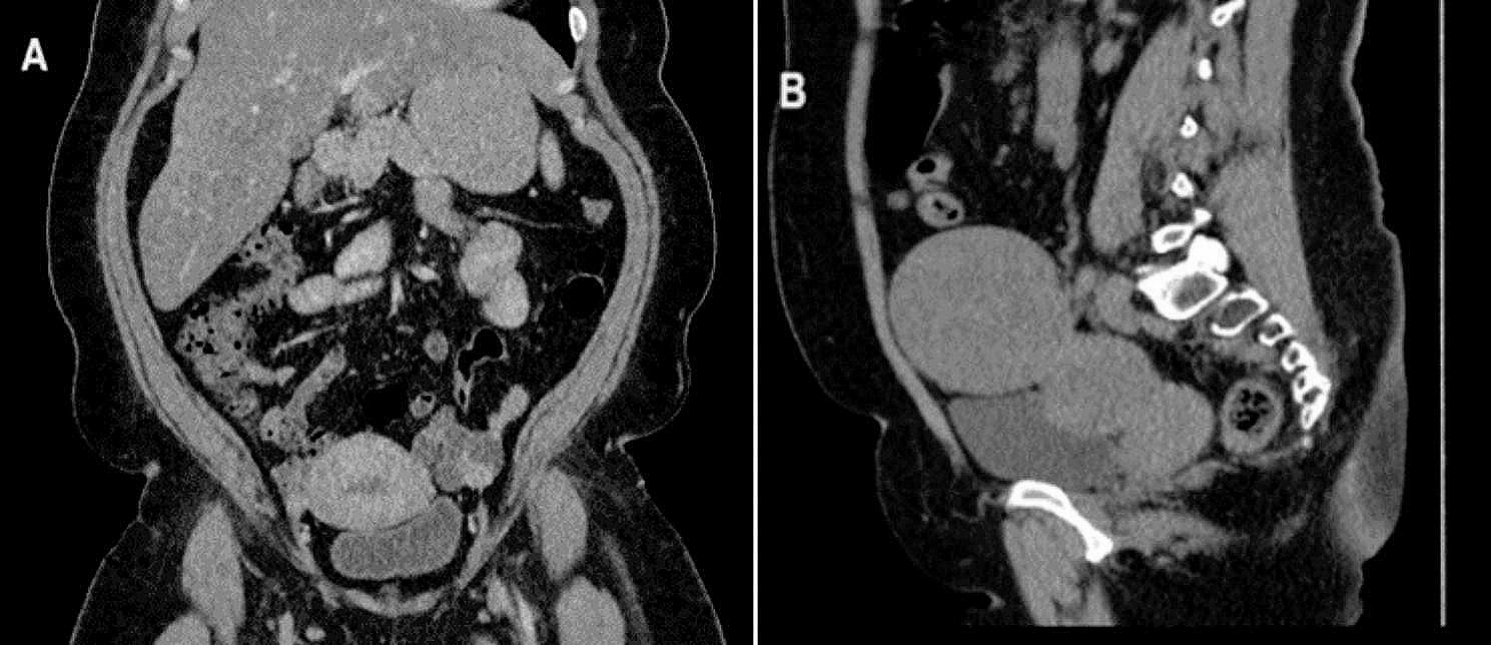
CT abdomen pelvis. (**A**) 3.8 × 2.7 × 4 cm complex left adnexal mass containing thick septations and enhancing soft tissue component. (**B**) 8.8 × 9 × 9.5 cm well defined heterogenous soft tissue mass in relation to left adnexa

**Fig. 2:**
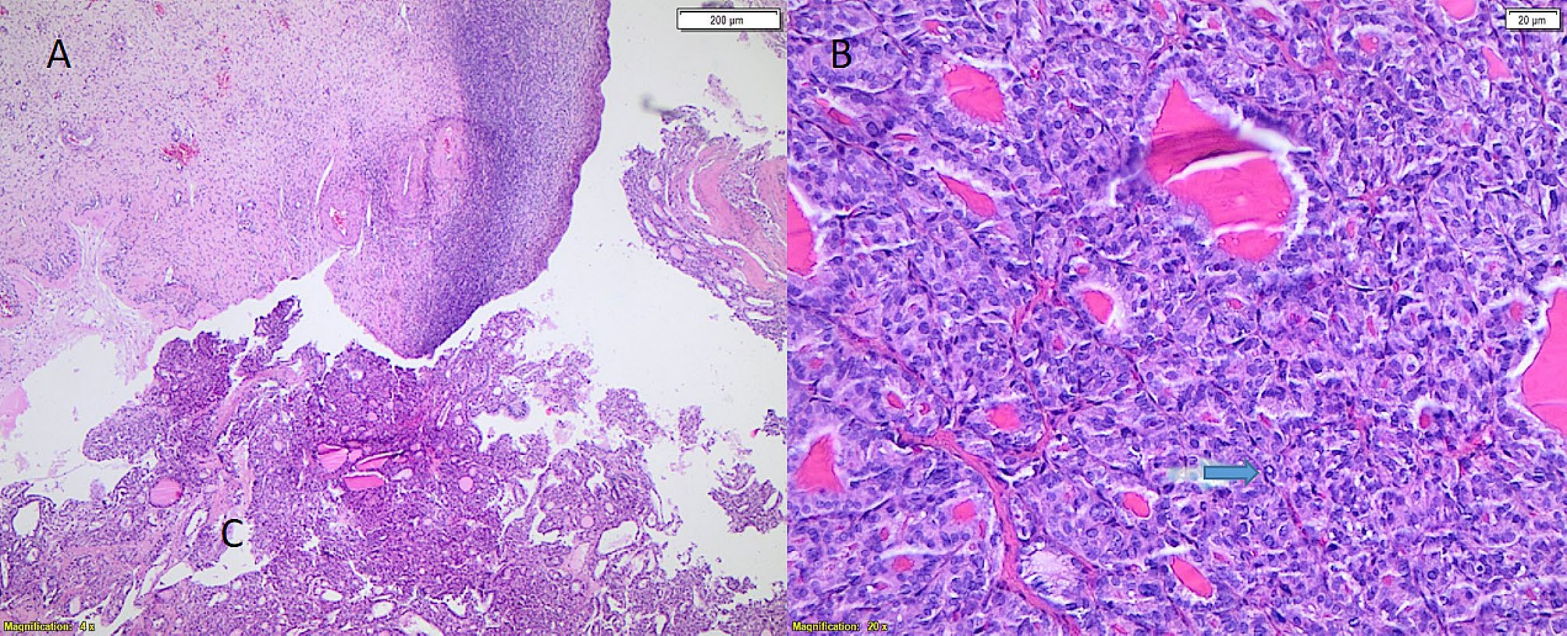
Microscopic appearance of the tumor, **A**. Thyroid tissue and adjacent ovarian stroma. **B.** Nuclear features of papillary carcinoma including nuclear enlargement, irregular nuclear membrane, chromatin clearing and occasional nuclear pseudo inclusions (arrow) (A Hematoxylin-eosin stain x40), (B, Hematoxylin-eosin stain x200)

**Fig. 3:**
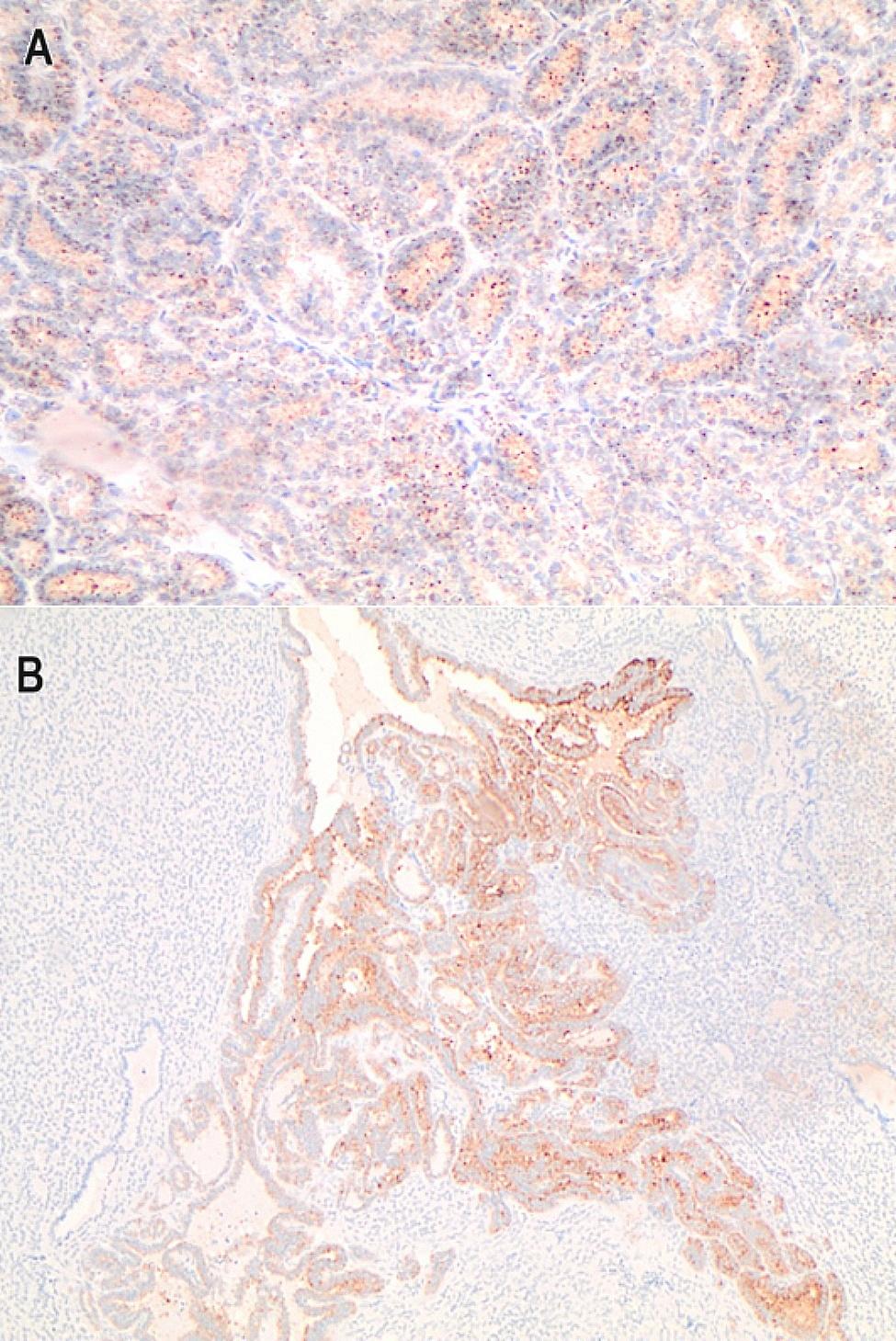
BRAF V600E IHC stain. (**A**) Case1, infiltrative follicular subtype of papillary thyroid carcinoma (PTC) (20X). (**B**) Case 2, classic PTC (20X)

A 44-year-old multiparous female came to the outpatient gynaecological clinic complaining of an irregular period for 5 months. In the diagnostic workup, transvaginal ultrasound showed a bulky uterus and a 2 × 3 cm left ovarian echogenic lesion mostly a dermoid cyst, the right ovary was normal. Lab investigations revealed free thyroxine (fT4) of 11 pmol /L (reference interval: 12–30 pmol/L), free triiodothyronine (fT3) of 5.84 pmol/L (reference interval: 2–7 pmol/L), thyroid stimulating hormone (TSH) of 1.94 mIU/L (reference interval: 0.4 to 4.0 mIU/L) and creatinine of 50 µmol/L (reference interval: 53 to 97.2 µmol/L for women). The patient’s surgical history revealed a left ovarian cystectomy. CT chest abdomen and pelvic was performed and showed a 3.8 × 2.7 × 4 cm complex cystic lesion with thick septation and enhancing soft tissue component (Fig. 1-A). Tumour markers including CA125, CEA, AFP, CA19-9, and hCG were within normal range. Subsequently, the patient underwent laparoscopic left salpingo-oophorectomy, diagnostic hysteroscopy and dilatation and curettage in April 2021. Operative findings were a multiloculated left ovarian cyst with both cystic and solid components, otherwise normal uterus, right ovary and tube, omentum and normal pelvic and abdominal peritoneal cavity. The left ovary was removed intact and retrieved through a bag without any spillage to the peritoneal cavity. Histopathology of the left ovarian cyst showed only thyroid tissue within ovarian stroma (struma ovarii). There was a focus (1.0 cm) in which tumor cells are arranged in infiltrative follicular pattern with no definite papillary formations; these cells exhibit typical nuclear features of papillary thyroid carcinoma (PTC). There was no necrosis or increased mitotic activity (up to 3 mitoses/HPF) and granular cytoplasmic staining for BRAF V00E immunohistochemical (IHC) stain was positive (Figs. 2 and 3). Endometrial biopsy was normal with no evidence of hyperplasia or malignancy. Cytology of peritoneal fluid was positive for involvement by carcinoma. After surgery, anti-thyroglobulin level was performed and was normal. The case was discussed at our multidisciplinary meeting and the plan was to perform total thyroidectomy followed by radioactive iodine therapy and then a hysterectomy with right salpingo-oophorectomy. She had a total thyroidectomy on the 26 of April 2021 and the pathology showed normal thyroid tissue with two small hyperplastic nodules with no evidence of malignancy. The postoperative course was uncomplicated. Later, the patient had radioactive iodine therapy (120 mCi of I131, oral). Post-iodine ablation whole body scan revealed functioning remnant thyroid tissue in the thyroid bed and no evidence of metastatic thyroid cancer. The patient was started on thyroxine and a total laparoscopic hysterectomy and right salpingo-oophorectomy was performed in July 2021. The uterus, cervix and right ovary and tube were all negative for tumour. Currently, the patient is doing well and she is under long-term (10 years) follow-up and monitoring with yearly serum thyroglobulin. She remains free of recurrence to this date.

## Case 2

**Fig. 4:**
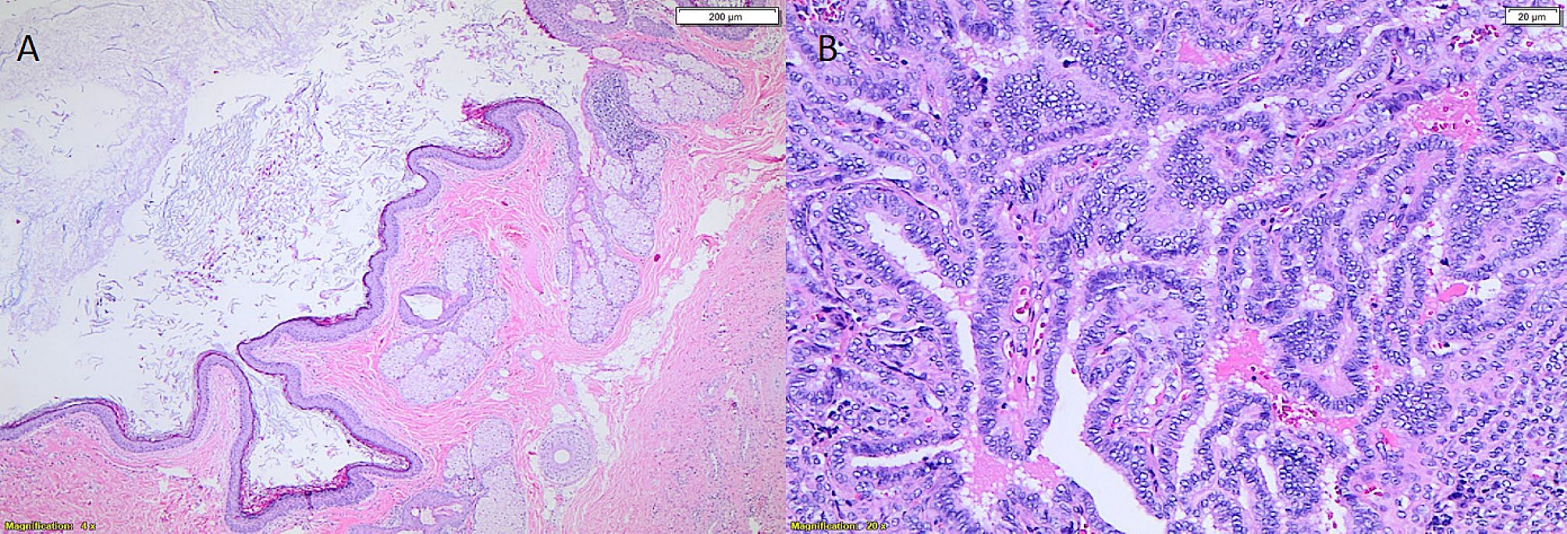
Microscopic appearance of the tumor. (**A**) The ectodermal component of the ovarian teratoma including skin and adnexal structures. (**B**) Nuclear features of papillary carcinoma, true papillae, nuclear enlargement, irregular nuclear membrane, and chromatin clearing (A Hematoxylin-eosin stain x40), (B, Hematoxylin-eosin stain x200)

A 46-year-old single female presented to the hospital with left lower abdominal pain that persisted for 2 months. The pain was localized without any radiation. Her period was regular and average in amount. The patient had hyperlipidemia and a family history of colon cancer. An ultrasound revealed a bulky uterus, intramural fibroid (1*2 cm), and 9 × 8 cm left ovarian cystic lesion with echogenic small projections in the cyst wall. The right ovary was normal. Tumour markers including CA125, CEA, Ca19-9 and hCG were within normal range. A thyroid function test was performed and was normal; TSH was 3.5 mIU/L and fT4 was 14 pmol/L. CT abdomen and pelvis were performed and showed a 9 × 9.5 × 8 cm well-defined heterogeneous left ovarian soft tissue mass containing tiny foci of calcification (Fig. 1-B). The patient underwent laparoscopic left salpingo-oophorectomy in July 2022 and was discharged without any complications. During surgery, the left ovary was removed intact and retrieved through a bag without any spillage to the peritoneal cavity. The histopathology revealed a mature cystic ovarian teratoma. Thyroid tissue with a central focus (0.5 cm) in which well-formed papilla with typical PTC nuclear features was identified. There was no evidence of necrosis or increased mitotic activity (up to 3 mitoses/HPF). The histopathology showed positive granular cytoplasmic staining for BRAF V00E immunohistochemical (IHC) stain (Figs. 3 and 4). Peritoneal cytology was negative for malignancy. Post-operative CT neck, chest, abdomen and pelvis were performed and showed no evidence of metastasis, however, the thyroid gland showed homogeneous enhancement with a small hypodense nodule seen in the left thyroid lobe measuring about 0.8 × 1 cm. A thyroid ultrasound was performed and showed a homogeneous thyroid gland containing few nodules seen on the left lobe, the largest nodule measuring about 1.4 × 1.3 × 1.2 cm. A few sub-centimetric deep cervical and submandibular lymph nodes were also noted bilaterally. The case was discussed at our multidisciplinary team meeting and the decision was to proceed with thyroidectomy followed by radioactive iodine and hysterectomy with right salpingo-oophorectomy to be considered later. The patient underwent total thyroidectomy in October 2022 and the histopathology report showed a multinodular goitre with no evidence of malignancy. This was followed by radioactive iodine ablation (136 mCi I131, oral). A subsequent iodine whole-body scan revealed no sites of iodine-avid metastatic disease. The patient was started on thyroxine and she has total abdominal hysterectomy and right salpingo-oophorectomy in July 2023.

## Discussion

SO is a highly specialized form of mature ovarian teratoma consisting of thyroid tissue showing all histological features of the thyroid gland. Depending on the histologic features, struma ovarii can be classified as benign or malignant; the diagnostic criteria for MSO are similar to those for differentiated thyroid cancer [4, 10, 11, 14]. Features indicative of malignancy include cytologic atypia, nuclear grooves, “ground-glass” overlapping nuclei, increased mitotic activity, and vascular invasion [4, 10]. Based on WHO 2022 classification of thyroid neoplasm, malignant follicular cell-derived neoplasms are stratified based on molecular profiles (BRAF V600E–like and RAS like tumors) and aggressiveness. Classic PTCs is defined by well-formed papillae lined by tumor cells with nuclei showing a specific set of nuclear features: enlargement, peripheral margination of chromatin, clearing of nucleoplasm, irregular contours forming grooves, and resulting in cytoplasmic pseudo inclusions. Infiltrative follicular variant PTC has predominant follicular architecture with florid nuclear atypia. It has infiltrative growth pattern of classic PTC but lacks prominent papillae [15]. BRAF V600E is the most common molecular alteration in PTC [15]. Jason Schmidt et al., showed that the development of MSOs with PTC features is associated with BRAF mutations of the type commonly observed in PTC, suggesting a common pathogenesis for all PTCs regardless of location. In contrast, mutations in the RET/RAS/RAF pathway are not found in BSO [16]. 

MSO represented less than 5% of SO cases and the most common type was papillary thyroid carcinoma [4, 10–12]. About 6–27% of MSO disseminates overall and the predominant sites of metastasis were adjacent pelvic structures, including the contralateral ovary. Distant metastases, including metastases to the lungs, bone, liver, and brain had been reported [9, 11, 12, 17, 18]. Only 6% of MSO cases are bilateral and tumours occur more commonly in the left ovary than in the right ovary [1, 11]. There are no standardized diagnostic criteria for MSO and is often diagnosed after surgery.

Due to the rarity of MSO, optimal management has not yet been defined. Surgical management of the primary tumour (cystectomy, USO or hysterectomy with BSO) and the potential indications for total thyroidectomy and radioactive iodine therapy (RAI) have not been standardized [7, 10, 13, 17]. DeSimone et al. reported that more recurrence occurred in patients treated conservatively with pelvic surgery only than in those who received additional surgical or radioiodine treatment [11]. Similarly, Jane et al. reported a recurrence of 21% in 42 cases treated with surgery only [12]. Cui et al. reported a 1.5% reduction in the mortality rate among patients treated with RAI compared with patients who did not receive RAI; however, the difference was not statistically significant (*p* > 0.05).^4^ Marti et al. suggested that pelvic surgery alone would be sufficient, provided there is no evidence of gross extra-ovarian extension and extensive pelvic surgery with prophylactic total thyroidectomy and RAI therapy may be reserved for patients with gross extra-ovarian extension or distant metastases [10]. Other authors suggested that risk stratification of MSO similar to that used in thyroid carcinoma can help determine the need for adjuvant treatment; [7, 12, 13, 17] patients with MSO confined to the ovary, measuring < 2 cm, of papillary histology without aggressive histopathological features can be considered low risk and thus only pelvic surgery followed by thyroxine is recommended [7, 12, 13, 17]. Conversely, for patients with known distant metastases and/or gross extra-ovarian extension of the tumour, tumour larger than 2 cm, histology other than papillary carcinoma, the presence of a *BRAF* mutation, or a synchronous primary thyroid cancer, complete thyroidectomy and adjuvant RAI treatment is recommended [7, 13, 17]. Furthermore, thyroxin therapy was also recommended by some authors in both low and high-risk patients to maintain the TSH levels in the 0.1 to 0.5 mU/L range for the first five years and then in the normal range thereafter in patients with no evidence of disease similar to that for patients with thyroid carcinoma [7, 13, 17]. In young women with MSO who wish to preserve fertility, MSO could be treated with conservative surgery such as unilateral oophorectomy if there is no evidence of extra-ovarian extension or distant metastasis [11, 17–19]. 

Recurrence rates of MSO vary; in a study by Marti et al., reporting a follow-up of 57 cases for 25 years, the recurrence rate was 7.5%.^10^ However, DeSimone et al. reported a recurrence rate of 35%.^11^ The average time to recurrence was approximately 4–6 years, [7, 12, 16] however, cases of late recurrence have been described [7, 10]. and thus, similar to thyroid carcinoma, long-term (10 to 20 years) follow-up by clinical examination and monitoring thyroglobulin level has been recommended by many authors [7, 12, 13, 18]. Thyroglobulin is a sensitive tumour marker that can be used for monitoring women with MSO. Thyroglobulin is a protein precursor of thyroid hormones, synthesized and secreted by the thyroid follicular cells [7]. Thyroglobulin becomes undetectable in patients who had a complete thyroidectomy and RAI therapy; however, in low-risk patients without thyroidectomy, it is unlikely that thyroglobulin becomes undetectable. In such cases, recurrence will be suspected in cases with increasing thyroglobulin levels above baselines [4, 7, 17]. 

The survival outcomes and prognostic factors of MSO have not been well-defined due to its rarity. Sijian et al. demonstrated an excellent survival outcome in patients with MSO irrespective of the treatment strategy. Advanced stage disease (stage IV), age more than 55 years and poorly differenced tumour were independent risk factors of prognosis in patients with MSO [14]. Similarly, Cui et al. reported a 4.72% mortality rate among 127 patients and most of the death occurred in patients with distant metastasis [4]. 

Finally, Tzelepis et al. highlighted after completing their review that MSO can sometimes coexist with thyroid carcinomas which unfortunately are aggressive cancers [5]. Therefore, clinical evaluation and imaging of the thyroid are recommended for the possibility of concurrent thyroid cancer.

## Conclusion

MSO is a rare tumour and there are currently no guidelines for the management of this type of cancer. Treatment decisions must be made individually based on clinical and pathological data and a multidisciplinary approach. In low-risk patients (tumours confined to the ovary, smaller than 2 cm and of papillary histology without aggressive histopathological features), pelvic surgery with/without thyroxine therapy is recommended. For those patients with a higher risk of recurrence, thyroidectomy with RAI therapy and thyroxine therapy are indicated. The prognosis is closely related to patient’s age, type of cancer, and the presence of distal metastasis. Similar to thyroid cancer, long-term (at least 10 years) follow-up is recommended for women with MSO.

### Electronic supplementary material

Below is the link to the electronic supplementary material.

Supplementary Material 1

## Data Availability

The datasets used and/or analysed during the current study available from the corresponding author on reasonable request.
