# Supplementary material for: Presentation and treatment of two cases of malignant struma ovarii
Source: BMC Womens Health. 2024 Mar 5;24:158. doi: 10.1186/s12905-024-03002-5 (PMC10913386; doi:10.1186/s12905-024-03002-5)
Supplement: Supplementary file 1 — Supplementary Material 1 [file 12905_2024_3002_MOESM1_ESM.pdf]

CARE guidelines for case reports: 13-item checklist

Please indicate in which section each item has been reported in your manuscript. If you feel that an item does not apply to your manuscript, please enter N/A.

For more information about the CARE guidelines, please see http://www.care-statement.org/.

No.	Description	Section #
**Title**		
1	The area of focus and "case report" should appear in the title	P4/L1
**Keywords**		
2	Two to five key words that identify topics covered in this case report	P5/L30
**Abstract**		
3a	Introduction—What is unique about this case? What does it add to the medical literature?	P4/L5
3b	The main symptoms of the patient and the important clinical findings	P4-5/L8-23
3c	The main diagnoses, therapeutics interventions, and outcomes	P4-5/L8-23
3d	Conclusion—What are the main 'take-away' lessons from this case?	P5/L25
**Introduction**		
4	Briefly summarize why this case is unique with medical literature references	P5-6/L35-55
**Patient information**		
5a	De-identified demographic information and other patient specific information	N/A
5b	Main concerns and symptoms of the patient	P6/L61 and P8/L98
5c	Medical, family, and psychosocial history including relevant genetic information	P8/L99
5d	Relevant past interventions and their outcomes	P7/L67
**Clinical findings**		
6	Describe the relevant physical examination (PE) and other clinical findings	N/A
**Timeline**		
7	A timeline of relevant information from the patient's history and this episode of care	P6-9/ eg, L72, L86
**Diagnostics assessment**		
8a	Diagnostic methods (such as PE, laboratory testing, imaging, surveys)	P6-9/ eg, L64-69
8b	Diagnostic challenges (such as access, financial, or cultural)	N/A
8c	Diagnostic reasoning including a differential diagnosis	N/A
8d	Prognostic characteristics (such as staging in oncology) where applicable	P6-9/L69-70
**Therapeutic intervention**		
9a	Types of intervention (such as pharmacologic, surgical, preventive, self-care)	P7/L71 and P8/L106
9b	Administration of intervention (such as dosage, strength, duration)	P7/L88 and P8/l124
9c	Changes in intervention with rationale	P6-9/ eg, L116
**Follow-up and outcomes**		
10a	Clinician and patient-assessed outcomes when appropriate	Page 4-6
10b	Important follow-up diagnostic and other test results	P9/L124
10c	Intervention adherence and tolerability (how was this assessed?)	N/A
10d	Adverse and unanticipated events	N/A
**Discussion**		
11a	Discussion of the strengths and limitations in your approach to this case	P9-12
11b	Discussion of the relevant medical literature	P9-12
11c	The rationale for conclusions (including assessment of possible causes)	P9-12
11d	The primary "take-away" lessons of this case report	P12L208-216
**Patient perspective**		

12	When appropriate, the patient can share their perspective on their case	N/A
**Informed consent**		
13	The patient should give informed consent	P13/221

When submitting your manuscript via the online submission form, please upload the completed checklist as a Figure/supplementary file.

If you would like this checklist to be included alongside your article, we ask that you upload the completed checklist to an online repository and include the guideline type, name of the repository, DOI and license in the *Data availability* section of your manuscript.

Developed from: Riley DS, Barber MS, Kienle GS, AronsonJK, von Schoen-Angerer T, Tugwell P, Kiene H, Helfand M, Altman DG, Sox H, Werthmann PG, Moher D, Rison RA, Shamseer L, Koch CA, Sun GH, Hanaway P, Sudak NL, Kaszkin-Bettag M, Carpenter JE, Gagnier JJ. CARE guidelines for case reports: explanation and elaboration document. J Clin Epidemiol. 2017 May 18. pii: S0895-4356(17)30037-9. https://doi.org/10.1016/j.jclinepi.2017.04.026
